# Advanced cardiotoxicity profiling using field potential imaging with UHD-CMOS-MEA in human iPSC-derived cardiomyocytes

**DOI:** 10.1093/toxsci/kfaf134

**Published:** 2025-09-30

**Authors:** Naoki Matsuda, Nami Nagafuku, Kazuki Matsuda, Yuto Ishibashi, Tomohiko Taniguchi, Yusaku Matsushita, Norimasa Miyamoto, Takashi Yoshinaga, Ikuro Suzuki

**Affiliations:** Department of Electronics, Graduate School of Engineering, Tohoku Institute of Technology, Sendai, Miyagi 982-8577, Japan; Department of Electronics, Graduate School of Engineering, Tohoku Institute of Technology, Sendai, Miyagi 982-8577, Japan; Department of Electronics, Graduate School of Engineering, Tohoku Institute of Technology, Sendai, Miyagi 982-8577, Japan; Department of Electronics, Graduate School of Engineering, Tohoku Institute of Technology, Sendai, Miyagi 982-8577, Japan; Advanced Biosignal Safety Assessment, Biopharmaceutical Assessments Unit, Tsukuba, Ibaraki 300-2635, Japan; Advanced Biosignal Safety Assessment, Biopharmaceutical Assessments Unit, Tsukuba, Ibaraki 300-2635, Japan; Advanced Biosignal Safety Assessment, Biopharmaceutical Assessments Unit, Tsukuba, Ibaraki 300-2635, Japan; Advanced Biosignal Safety Assessment, Biopharmaceutical Assessments Unit, Tsukuba, Ibaraki 300-2635, Japan; Department of Electronics, Graduate School of Engineering, Tohoku Institute of Technology, Sendai, Miyagi 982-8577, Japan

**Keywords:** cardiotoxicity, human iPSC-derived cardiomyocytes, UHD-CMOS-MEA, field potential imaging, chronic cardiotoxicity, in vitro toxicity assessment

## Abstract

Accurate assessment of cardiotoxicity using human induced pluripotent stem cell (iPSC)-derived cardiomyocytes is critical for ensuring drug safety during preclinical development. However, existing in vitro methodologies predominantly focus on QT interval prolongation and arrhythmia risk, often lacking the capacity to capture the complex interplay among multiple ion channels or to detect early manifestations of chronic cardiotoxicity—both of which are essential for evaluating long-term cardiac safety. Moreover, reliable prediction of pharmacological mechanisms of action remains a significant challenge. In this study, we employed field potential imaging utilizing an ultra-high-density complementary metal–oxide–semiconductor microelectrode array (MEA) comprising 236,880 electrodes distributed across a 5.9 × 5.5 mm active area. With 91.9% surface coverage by 11 μm electrodes spaced at 0.25 μm, the platform achieves near single-cell resolution across the entire cardiomyocyte monolayer. This system enabled the extraction of high-resolution electrophysiological endpoints, including the number and spatial variability of excitation origins, conduction velocity, and propagation area—thereby extending the analytical capabilities beyond those of conventional MEAs. Pharmacological testing revealed compound-specific alterations: Isoproterenol increased excitation origins, mexiletine reduced conduction velocity, and E-4031 diminished propagation area. Although these agents are well characterized, their effects were visualized with unprecedented spatiotemporal resolution, reflecting their underlying mechanisms of action. Multivariate analysis incorporating both conventional and novel endpoints enabled accurate classification of mechanisms under acute conditions. Furthermore, chronic cardiotoxicity induced by low-dose doxorubicin (0.03 μM) was sensitively detected within 24 h—earlier and at lower concentrations than previously reported—based on significant reductions in conduction velocity and propagation area. Collectively, these findings establish a high-resolution, mechanism-aware framework for in vitro cardiotoxicity profiling, offering improved predictive accuracy by capturing multi-ion channel interactions, spatial conduction abnormalities, and early signs of chronic dysfunction.

Cardiotoxicity remains one of the most critical safety concerns in drug development and poses a significant threat to patient safety (Fung et al. 2001; Cook et al. 2014; Onakpoya et al. 2016; Mamoshina et al. 2021). In particular, drug-induced QT prolongation and Torsades de Pointes (TdP) arrhythmias are widely recognized as major risks, often leading to clinical trial termination or market withdrawal (Stockbridge et al. 2013). Historically, the prediction of cardiotoxicity has relied on hERG channel inhibition assays and animal models. The ICH S7B guideline played a pivotal role in standardizing the assessment of QT prolongation risk via hERG blockade and has served as a foundation for cardiac safety evaluation (The ICH Steering Committee 2005; Gintant 2011). However, this framework primarily focuses on hERG inhibition and lacks comprehensive coverage of mechanisms involving other ion channels such as Na^+^ and L-type Ca^2+^ channels (Sager et al. 2014; Colatsky et al. 2016). To overcome these limitations, the Comprehensive in vitro Proarrhythmia Assay (CiPA) initiative was introduced, integrating multi-ion channel assays, human induced pluripotent stem cell (iPSC)-derived cardiomyocyte (hiPSC-CM) models, and in silico simulations into a unified framework (Colatsky et al. 2016; Li et al. 2019). This approach not only evaluates QT prolongation but also aims to quantitatively predict TdP risk and has been adopted by regulatory agencies for drug safety evaluation. In parallel, hiPSC-CMs have garnered increasing interest as an in vitro platform for cardiotoxicity assessment (Kanda et al. 2018). Because hiPSC-CMs recapitulate key aspects of human electrophysiology, they offer the potential to detect toxicity risks that are not apparent in animal models (Ma et al. 2011; Hoekstra et al. 2012; Gibson et al. 2014; Blinova et al. 2017; Lee et al. 2024). However, accurately predicting cardiotoxic risk remains challenging for drugs that act on multiple ion channels due to the complex interplay and hierarchical contribution of these channels. For instance, drugs like verapamil and bepridil, which modulate hERG, ICaL, and INa, often show poor correlation between in vitro results and clinical observations (Blinova et al. 2018). Additionally, CiPA primarily targets acute toxicity and does not adequately address chronic cardiotoxicity.

To resolve these issues, more comprehensive and high-resolution methods are needed to evaluate both acute and chronic cardiotoxic effects, while accounting for complex multi-channel interactions. Several in vitro technologies have been developed, including impedance analysis, calcium transient imaging, and high-content imaging (Guo et al. 2013; Pointon et al. 2013; Doherty et al. 2015; Dempsey et al. 2016; Aikawa 2017; Lu et al. 2019). Although impedance assays are sensitive to morphological changes and cell–matrix interactions, and calcium imaging enables real-time monitoring of excitation–contraction coupling, neither method provides direct measurement of membrane potential or detailed spatial propagation of electrical activity. As a result, they are limited in their ability to fully capture the dynamics of ion channel interactions. In contrast, microelectrode array (MEA) enable non-invasive, multi-site recordings of extracellular potentials and have been widely used to assess conduction velocity and electrical remodeling in cardiomyocytes (Clements et al. 2015; Ando et al. 2017; Blinova et al. 2017, 2018; Kanda et al. 2018; Millard et al. 2018). However, traditional MEAs are constrained by sparse electrode configurations, typically relying on field potential duration measurements at a small number of discrete sites. To overcome these limitations in spatial resolution and data richness, the integration of large-area, high-density complementary metal–oxide–semiconductor (CMOS) technology has recently emerged as a promising approach (Yuan et al. 2021; Emery et al. 2022; Suzuki et al. 2023). This technology holds the potential to reveal previously undetectable functional characteristics of cardiomyocyte populations.

Building on this concept, we propose a novel high-resolution cardiotoxicity evaluation platform based on field potential imaging (FPI) using an ultra-high-density (UHD) CMOS-MEA comprising 236,880 electrodes. This system enables detailed mapping of action potential propagation across hiPSC-CM monolayers and allows for the extraction of novel electrophysiological endpoints—including the number and variability of excitation origins, conduction velocity, and propagation area. These features facilitate a more nuanced analysis of drug-induced electrophysiological changes, including mechanistic evaluation of multi-channel compounds and early detection of chronic toxicity.

In this study, we demonstrate that our approach effectively captures both acute and chronic cardiotoxic effects, as exemplified by doxorubicin-induced alterations. Furthermore, we show that the system allows mechanism-aware profiling of compounds such as bepridil, which are often misclassified as low-risk by conventional methods. By combining novel electrophysiological endpoints derived from FPI with multivariate analysis, this study provides a framework for mechanism-specific, spatiotemporal toxicity profiling that contributes to improved prediction and understanding of cardiotoxic risk in drug development.

## Materials and methods

### Chip preparation

MEA chips were first cleaned with Tergazyme solution (0.5 g in 50 ml sterile water at 60 °C). Each well was filled with 3 ml of the solution and incubated for 1 h at room temperature, followed by 4 rinses with distilled water. Next, 3 ml of 70% ethanol was added for 30 s, then washed again 4 times with DW. Chips were air-dried and UV-sterilized for 30 min in a biosafety cabinet. The dried chips were coated overnight at 4 °C with 10-fold diluted Type-C collagen (in 0.02 N acetic acid). After removing the solution, chips were air-dried at room temperature for 2 h. For enhanced cell adhesion, 15 µl of fibronectin solution (50 µg/ml in PBS) was applied to the electrode area and incubated at 37 °C for 1 h.

### Cell culture and plating

Cryopreserved iCell Cardiomyocytes (FUJIFILM Cellular Dynamics, Inc.) were handled according to the supplier’s instructions. Cells were thawed in a 37 °C water bath for approximately 3 min and resuspended in iCell Thawing Medium to prepare a cell suspension at a final concentration of 1 × 10^7^ cells/ml. A total of 10 µl of the cell suspension (100,000 cells per chip) was seeded directly onto the fibronectin-coated electrode area of each chip. Chips were incubated at 37 °C for 1 h to allow cell attachment, after which 1 ml of maintenance medium was gently added. The culture medium was replaced 24 h after seeding, with half the volume exchanged. Thereafter, medium exchanges were performed twice weekly by replacing half the medium volume.

### Immunocytochemistry

Immunocytochemistry was performed on hiPSC-CMs cultured on CMOS-MEA chips immediately after pharmacological experiments using the Stain Perfect Kit A (#SP-A-1000, Immusmol). Cells were incubated overnight at 4 °C with primary antibodies against MLC-2A (Synaptic Systems, 311011) and MLC-2V (ProteinTech, 10906-1-AP), both diluted 1:200 in the appropriate buffer. The following day, secondary antibodies—Goat anti-Mouse IgG (H + L) Alexa Fluor 488 (Invitrogen, A-11001) and Goat anti-Rabbit IgG (H + L) Alexa Fluor 546 (Invitrogen, A-11035)—were applied at a 1:1000 dilution. After 3 washes with Wash Solution 2, nuclei were stained with Hoechst 33258 (DOJINDO, 34307961) diluted 1:1000 for 10 min. Fluorescence images were acquired using a confocal laser scanning microscope (Nikon AX/AXR with NSPARK) equipped with a 20× objective lens.

### Pharmacological tests

Pharmacological tests were conducted using hiPSC-CMs between days in vitro (DIV) 7 and 10, during which spontaneous beating activity was consistently observed. All test compounds used in this study are listed in Table 1. Compounds were dissolved in culture medium containing 0.1% DMSO and administered cumulatively at increasing concentrations. Drug solutions were applied directly to the culture wells, and measurements were initiated 30 min after administration. To minimize variability due to medium exchange, the culture medium was replaced 1 day before testing, providing a 24-h equilibration period. All recordings were performed under spontaneous beating conditions, with no external pacing applied.

**Table 1.** Details of test compounds.

Compound	Class	Conc. (μM)	Source	CAS RN
Amoxicillin	β-lactam antibiotic	10, 100	A2099, Tokyo Chemical Industry Co.	61336-70-7
Aspirin	Analgesic, anti-pyretic	10, 100	A2093, Sigma-Aldrich	50-78-2
Azimilide	Class III antiarrhythmic	0.01, 0.1, 1	6318, Tocris Bioscience	149888-94-8
Bepridil	Anti-anginal	0.1, 1	B5016, Sigma-Aldrich	68099-86-5
Diltiazem	Class IV antiarrhythmic	0.1, 1, 10	047-20311, Fujifilm Wako Pure Chemical Co.	33286-22-5
Disopyramide	Class Ia antiarrhythmic	0.1, 1, 10	S5490, Selleck Chemicals	3737-09-5
E-4031	Class III anti-arrhythmic (research purposes only)	0.003, 0.03, 0.1	059-08451, Fujifilm Wako Pure Chemical Co.	N/A-05-0845-1
Isoproterenol	Class II anti-arrhythmic	0.01, 0.03	I0260, Tokyo Chemical Industry Co.	51-30-9
Mexiletine	Class Ib anti-arrhythmic	0.1, 1, 10	132-17581, Fujifilm Wako Pure Chemical Co.	5370-01-4
Quinidine	Class Ia antiarrhythmic	1, 3, 10	176-00111, Fujifilm Wako Pure Chemical Co.	56-54-2
Verapamil	Class IV anti-arrhythmic	0.003, 0.03, 0.1	222-00781, Fujifilm Wako Pure Chemical Co.	152-11-4
DMSO	Vehicle	0.1%	D2650, Sigma-Aldrich	67-68-5
Doxorubicin	Broad-spectrum anti-tumor	0.03, 0.1	D4193, Tokyo Chemical Industry Co.	25316-40-9

### Extracellular recording

Extracellular field potentials were recorded using an UHD CMOS-MEA system (Sony Semiconductor Solutions), equipped with 236,880 electrodes arranged over a 5.9 × 5.5 mm sensing area. Recordings were performed at a sampling rate of 2 kHz, with a 2-min recording duration per condition. Data acquisition was controlled via proprietary software (Sony Semiconductor Solutions), and all raw signals were stored for offline analysis. All experiments were conducted in a humidified environment at 37 °C with 5% CO_2_, ensuring physiological conditions during both drug exposure and recording.

### Conventional MEA recording

To enable comparison with the UHD-CMOS-MEA recordings, conventional extracellular field potential recordings were performed using 16-electrode MEA plates on a commercially available MEA system (Axion Maestro, Axion Biosystems), in accordance with the general principles of the CiPA initiative. hiPSC-CMs were cultured and maintained on the MEA plates under spontaneous beating conditions at 37 °C. Recordings were conducted after 10 to 14 DIV, and data acquisition and beat detection were performed using the built-in algorithms of the AxIS software (Axion Biosystems), without the use of external pacing. Pharmacological testing was carried out using 7 compounds commonly included in CiPA assessments: Azimilide, Bepridil, Diltiazem, E-4031, Mexiletine, Quinidine, and Verapamil. Each compound was applied under acute exposure conditions, and field potential recordings were obtained for 30 min following drug administration.

### Spike and beat detection

Spikes from hiPSC-derived cardiomyocytes were initially detected at each electrode using a voltage threshold of ±100 µV via BinViewer software (SCREEN Holdings Co., Ltd, Japan). The resulting spike data were then processed using a custom MATLAB (MathWorks, Natick, MA) script to perform beat detection. Firing count histograms were generated using a bin size of 0.5 ms, and peak detection within these histograms identified the timing and propagation duration of beats across the electrode array. For each electrode, the maximum spike amplitude and its corresponding peak timing were calculated to generate heat maps of field potential amplitude and activation timing.

### Statistical analysis

One-way ANOVA was performed for each of the 17 calculated beat parameters to assess whether the values at each concentration differed significantly from the pre-treatment baseline. For mechanism-of-action classification and the cardiotoxicity assessment of doxorubicin, a one-way multivariate analysis of variance (MANOVA) was employed, using principal component (PC)1 and PC2 scores as dependent variables to evaluate significant differences between drug mechanism groups. Statistical significance was determined at *P* < 0.05.

### Principal component analysis and cardiotoxicity assessment

To evaluate compound-specific cardiotoxicity risk, we conducted principal component analysis (PCA) on 17 beat-related electrophysiological parameters extracted from each recording. A total of 65,518 parameter combinations, selecting between 2 and 8 parameters, were systematically analyzed using the PCA function in MATLAB. Each parameter set was evaluated for its ability to separate cardiotoxic and non-cardiotoxic compounds while maintaining minimal variance among negative controls. The optimal set of 4 parameters—beat rate, interbeat interval (IBI), propagation area, and short-term variability (STV) of conduction velocity—was selected based on maximum separability and no significant difference among negative control groups (aspirin and amoxicillin) in one-way MANOVA applied to the first 2 PCs. To assess cardiotoxicity, PCA scores of test compounds were plotted in the PC space, and their positions were compared with the SD-defined zones of the negative control group. Risk levels were classified as follows: Low risk (within 1 SD), medium risk (between 1 and 2 SD), and high risk (beyond 2 SD). Dose-dependent cardiotoxicity potential was estimated based on displacement from the negative control cluster.

## Results

### Results 1. FPI of human iPSC-derived cardiomyocytes using 236,880-electrode UHD-CMOS-MEA measurements

**Fig. 1.**
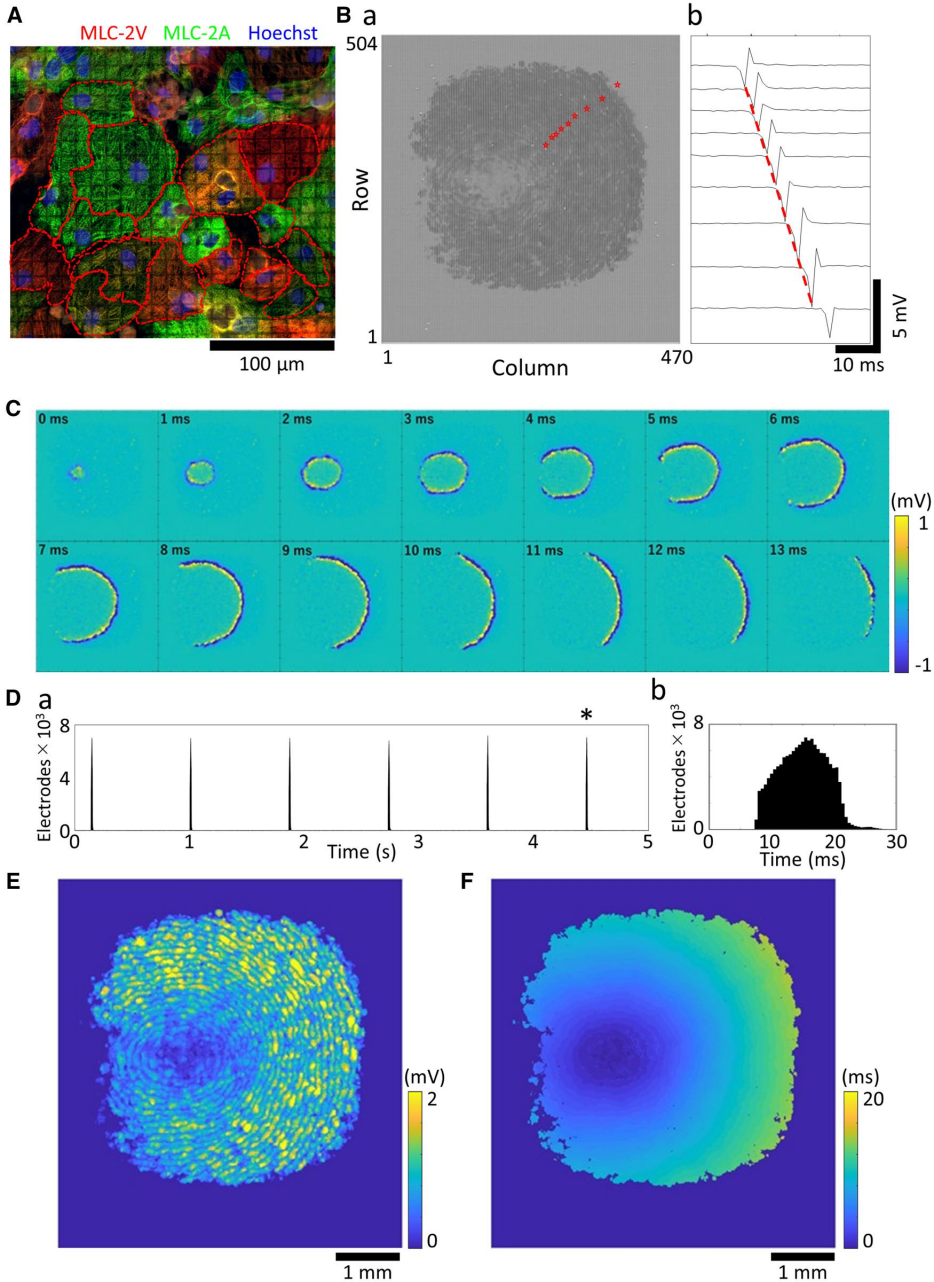
Field potential imaging in human iPSC-derived cardiomyocytes. A) Immunostaining images. Red: Myosin Light Chain 2 Ventricular (MLC-2V); Green: Myosin Light Chain 2 Atrial (MLC-2A); Blue: Hoechst 33258. B) a) Extracellular potential waveforms recorded from 236,880 electrodes. b) Extracellular potential waveform from the electrode marked with a star. C) Time-lapse of the potential heatmap for each sampling point across all electrodes during a single beat. D) a) Histogram of the number of active electrodes over 5 s (bin size = 0.5 ms). b) Enlarged view of a single beat marked with an asterisk in a). E) Heatmap of the maximum voltage amplitude during a single beat. F) Delay map of voltage peak timing during a single beat.

hiPSC-CMs were cultured on a CMOS-MEA chip for 7 to 10 DIV, and FPI was performed to record their spontaneous electrical activity. The CMOS-MEA used in this study comprises 236,880 electrodes arranged over a 5.9 × 5.5 mm active area, with 91.9% of the surface occupied by 11 μm electrodes spaced at 0.25 μm intervals. This configuration allows simultaneous recording of extracellular potentials from a wide area of the cardiomyocyte monolayer. Phase-contrast microscopy confirmed that the cells adhered uniformly to the electrode array and that individual cardiomyocytes spanned multiple electrodes (Fig. 1A). Spontaneous extracellular field potentials were recorded at a sampling frequency of 2 kHz, revealing widespread signal propagation across the cultured monolayer (Fig. 1B). Figure 1B-a shows the overall signal acquisition area, whereas Fig. 1B-b presents a representative waveform from a single electrode, with peak voltage measured at 1.98 ± 0.20 mV (*n* = 9 electrodes). Time-lapse analysis of potential heatmaps at each sampling point demonstrated that a single beat propagated across the monolayer within approximately 13 ms (Fig. 1C, Movie S1). Active electrodes were identified using a voltage threshold, and their temporal activation pattern was analyzed by binning the number of active electrodes in 0.5 ms intervals (Fig. 1D-a). This analysis revealed that each beat activated an average of 115,892 ± 50 electrodes (*n* = 6 beats, Fig. 1D-b). IBIs and beat counts were also derived from this histogram. The mean beat rate was 57.8 ± 1.7 bpm, and the average IBI was 1078.6 ± 29.8 ms across 54 wells (Fig. 1D). Additionally, two-dimensional heatmaps were generated: One displaying the peak amplitude of each electrode and another showing the timing of peak voltage. These visualizations illustrated the initiation sites, propagation direction, and spatial coverage of each beat (Fig. 1E).

### Results 2. Establishment of novel endpoints for beat propagation and detection of drug responses

To characterize the spatial and temporal dynamics of cardiomyocyte network activity, FPI was conducted using an UHD CMOS MEA. From the recorded extracellular field potentials, 3 electrophysiological parameters were extracted: Excitation origin count, conduction velocity, and propagation area.

**Fig. 2.**
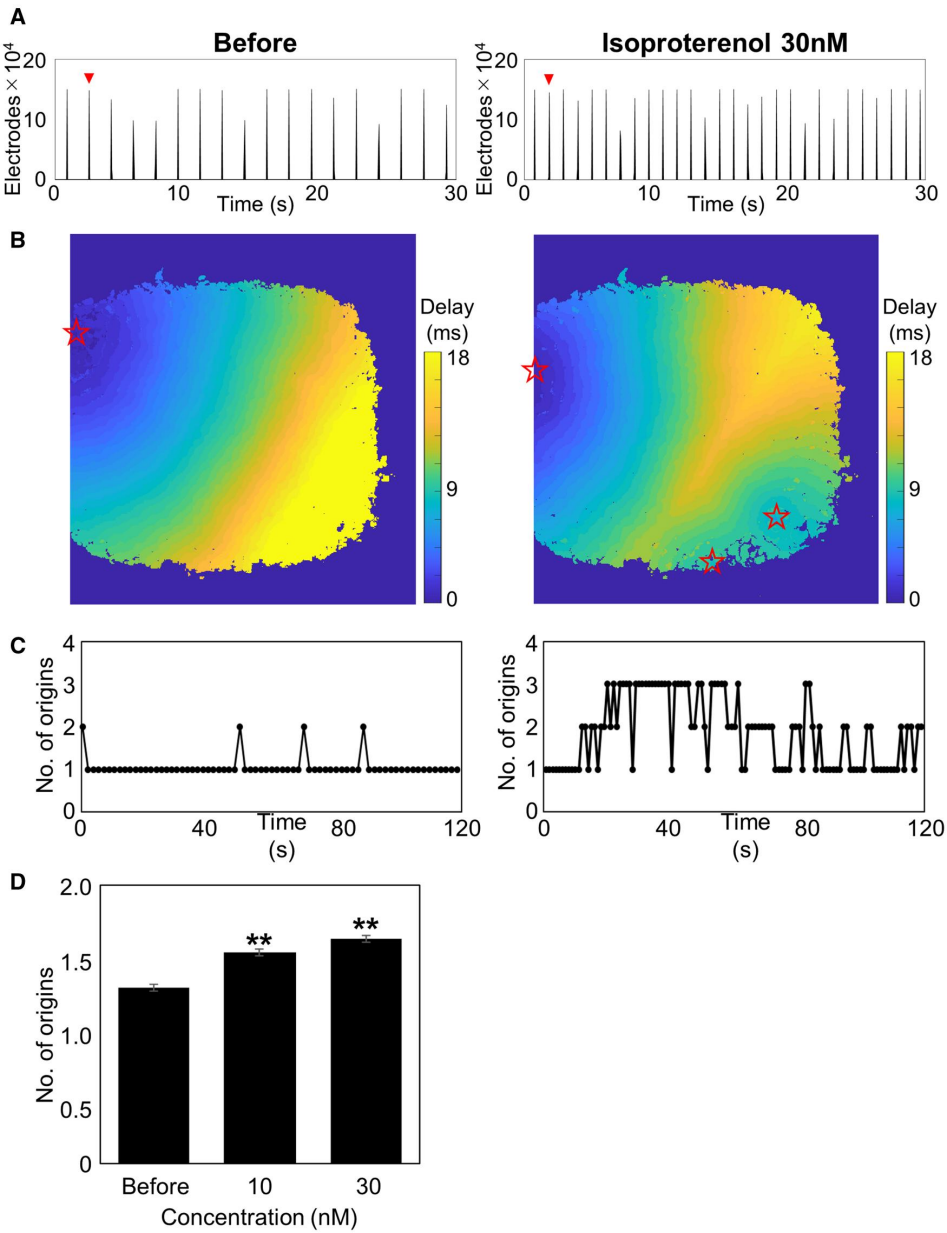
Increase in the number of excitation origins induced by isoproterenol. A) Histogram of the number of active electrodes over 30 s before (left) and after the treatment of 30 nM Isoproterenol (right). B) Delay map of voltage peak timing for a single beat, where stars indicate excitation origins. C) Plot of the number of excitation origins detected over a 2-min period. D) Concentration-dependent increase in the average number of excitation origins (*n* = 6 wells, **P < 0.01 vs. before).

Excitation origin count was determined by identifying the earliest activated electrode clusters during each beat using heatmaps based on peak field potential timing. Under control conditions, most beats originated from a single consistent location. After treatment with isoproterenol, the total number of active electrodes remained similar (Before: 149,149.4 ± 3,090.5; 30 nM: 149,185.2 ± 2,882.6), whereas the beating frequency increased from 35.5 to 57.0 bpm (Fig. 2A). Time-lapse heatmaps revealed that initial beats originated from the upper left region, whereas additional initiation sites emerged in the lower right region following isoproterenol administration (Fig. 2B). Cumulative plots over a 120-s period indicated an increased number of initiation sites after drug treatment (Fig. 2C). The average number of excitation origins per beat increased to 1.51 ± 0.02 at 10 nM and 1.61 ± 0.02 at 30 nM (*n* = 6 wells, ≥626 beats; Fig. 2D; Movie S2).

**Fig. 3.**
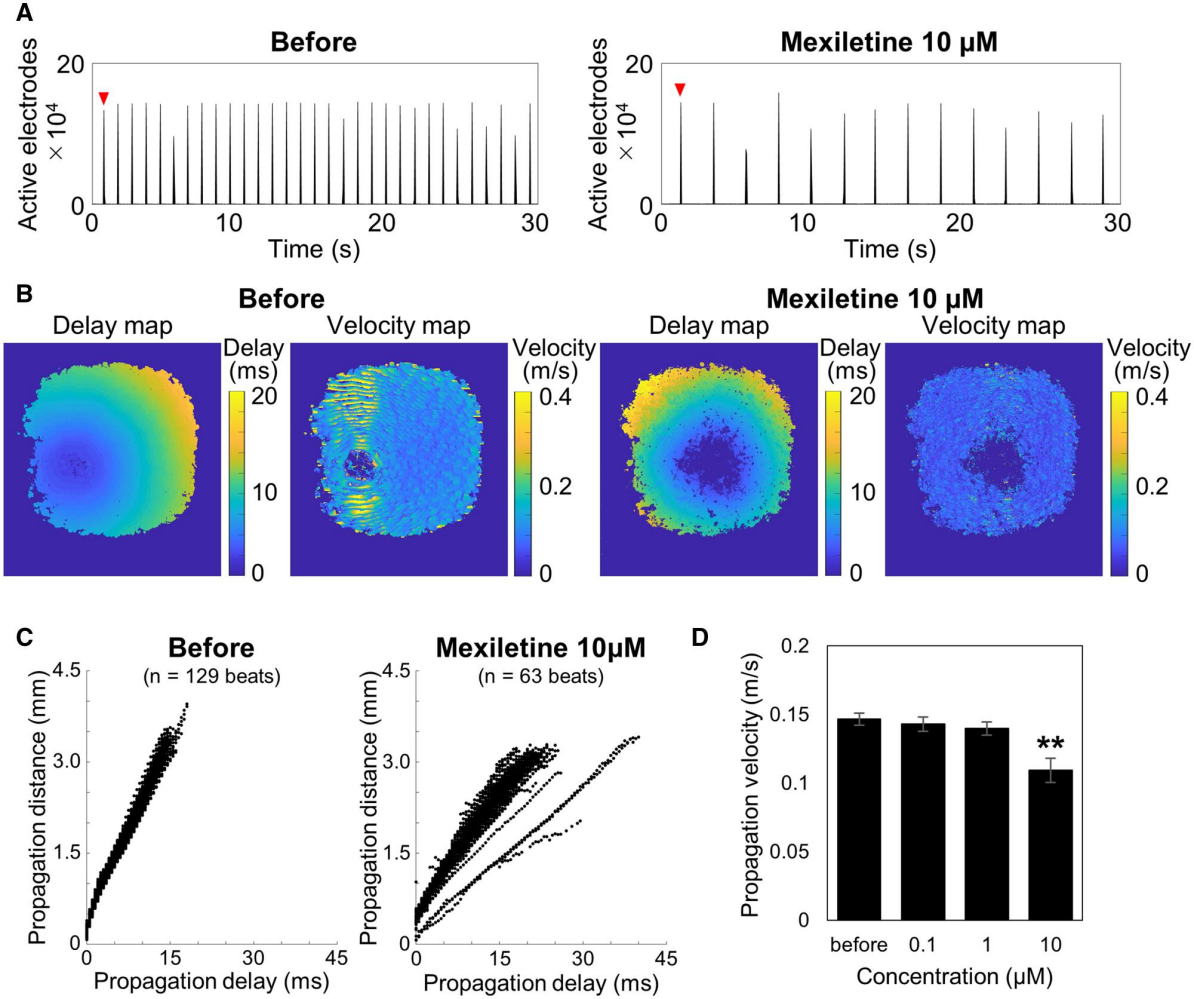
Decrease in propagation velocity induced by mexiletine. A) Histogram of the number of active electrodes over 30 s before (left) and after the treatment of 10 μM Mexiletine (right). B) Delay map of voltage peak timing and propagation velocity heatmap for a single beat. C) Plot of propagation time and propagation distance detected over a 2-min period. D) Concentration-dependent decrease in the average propagation velocity (*n* = 6 wells, **P < 0.01 vs. before).

Conduction velocity was calculated based on the relationship between conduction distance and time from the initiation site to each electrode. The overall conduction velocity was estimated from the slope of a linear regression, and local velocities were computed from electrodes with time differences of ±0.5 ms. After treatment with 10 µM mexiletine, the number of active electrodes decreased from 115,437.9 ± 582.1 to 93,595.6 ± 11,323.1, and the beating frequency decreased from 64.5 to 31.5 bpm (Fig. 3A). Heatmaps showed that the proportion of the area with conduction velocity exceeding 0.3 m/s declined from 4.9% to 0.6% (Fig. 3B). Beat-wise analysis revealed increased variability in conduction speed post-treatment (Fig. 3C), and the average conduction velocity decreased from 0.147 ± 0.004 to 0.109 ± 0.009 m/s (*n* = 6 wells; Fig. 3D; Movie S3).

**Fig. 4.**
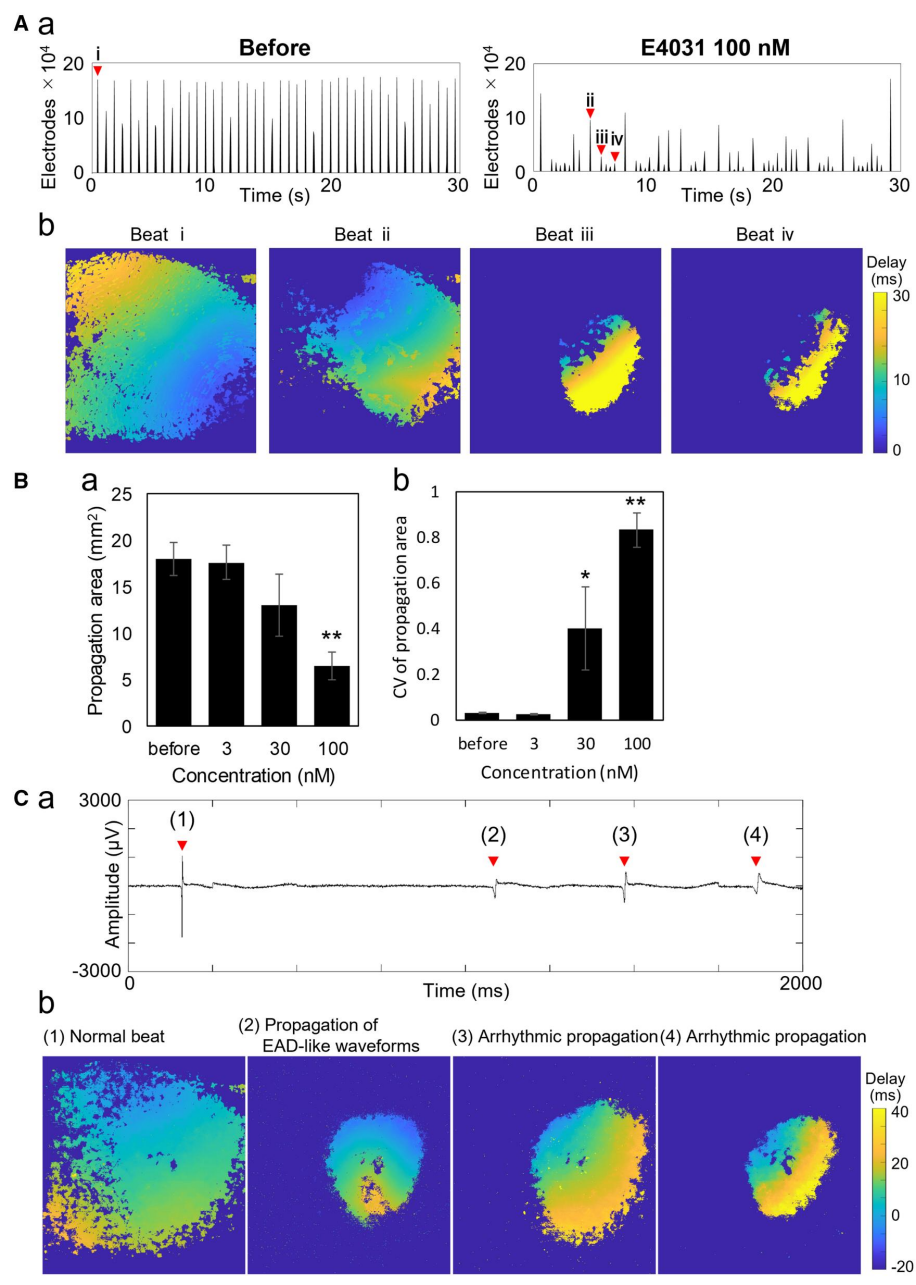
Decrease in propagation area and detection of EAD-like waveforms induced by E-4031. A) a) Histogram of the number of active electrodes over 30 s before (left) and after the treatment of 100 nM E-4031 (right). A) b) Heatmap of peak timing for each beat indicated by arrows in a). B) a) Concentration-dependent decrease in the average propagation area. B) b) Concentration-dependent increase in the coefficient of variation of propagation area (*n* = 6 wells). *P < 0.05 vs. before, **P < 0.01 vs. before. C) a) Field potential waveforms recorded after the treatment of 100 nM E-4031. C) b) Heatmap of peak timing for each beat indicated by arrows in a). 1) Beats spreading across the entire recording area. 2) Locally confined beats with EAD-like waveforms. 3) Locally confined arrhythmic propagation. 4) Locally confined arrhythmic propagation with prolonged propagation time.

Propagation area was evaluated as the number of electrodes that detected field potentials during each beat. Treatment with 100 nM E-4031 did not markedly change beat frequency (Before: 88 bpm; After: 82 bpm) but substantially reduced the number of active electrodes from 166,785.2 ± 4,264.5 to 43,675.2 ± 42,039.4 (Fig. 4A-a). Heatmaps showed that while full-area propagation occurred before treatment, propagation after treatment was often restricted to localized regions that varied between beats (Fig. 4A-b). The mean propagation area decreased from 17.98 ± 1.78 to 6.51 ± 1.51 mm^2^, and the coefficient of variation (CV) increased from 0.029 ± 0.004 to 0.400 ± 0.182 at 30 nM (*n* = 5 wells; Fig. 4B). At this concentration, partial beats were frequently observed following widely propagated beats. Field potential waveforms associated with these events exhibited delayed and spatially confined propagation (Fig. 4C-a), and the corresponding heatmaps confirmed localized activation patterns (Fig. 4C-b; Movie S4).

These newly characterized electrophysiological parameters, quantified using FPI of hiPSC-CMs, exhibited distinct response profiles to compounds with differing pharmacological actions.

### Results 3. Detection of acute cardiotoxicity and classification of mechanisms of action based on beat parameters

**Fig. 5.**
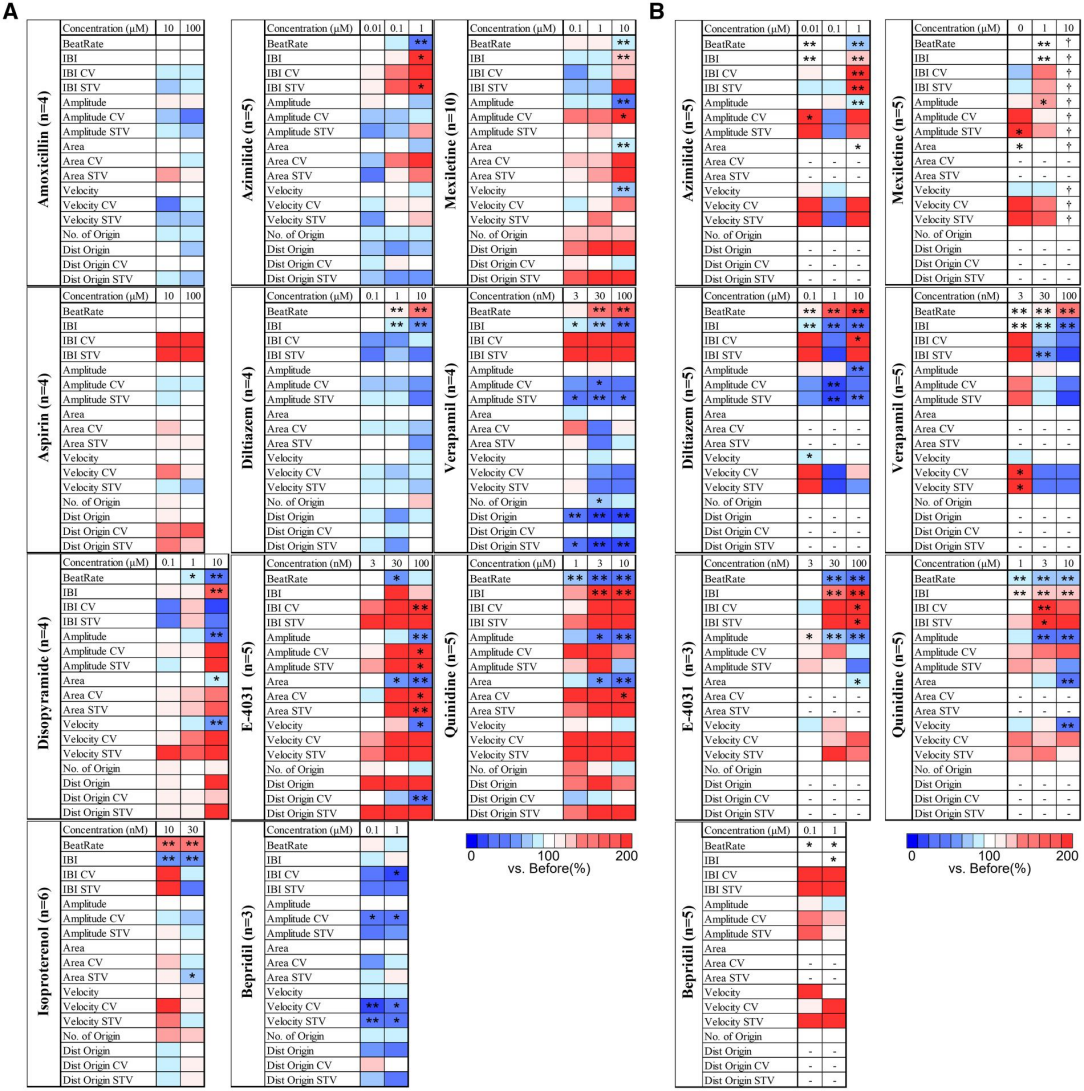
Heatmaps of beating parameter changes in the acute response to cardiotoxic and non-cardiotoxic compounds in human iPSC-derived cardiomyocytes. A) UHD-CMOS-MEA recordings for 11 compounds, showing changes in 17 beating-related parameters across multiple concentrations. B) Conventional MEA results for the corresponding compounds. Each value is normalized to 100% based on pre-treatment values for each parameter. One-way ANOVA followed by Dunnett’s post hoc test was performed. *P < 0.05 vs. before, **P < 0.01 vs. before. “—” indicates parameters not calculable due to insufficient spatial resolution in the conventional MEA system. “†” indicates values not available due to complete cessation of spontaneous beating after compound administration.

To assess the electrophysiological effects of acute drug treatment, FPI was conducted using CMOS-MEA on 11 compounds, including 9 with reported cardiotoxicity and 2 negative controls. A total of 17 beat-related parameters were extracted for analysis (Table 2). A heatmap summarizing the parameter profiles for each compound is shown in Fig. 5A.

**Table 2.** Description of analytical parameters.

Analytical parameter	Description
Beat rate (BPM)	Number of peaks in the histogram of the active electrodes
Interbeat interval (IBI, ms)	Average peak interval of histogram of active electrodes
CV of IBI	Coefficient of variation of IBI
STV of IBI	Short-Term Variance of IBI
Amplitude (mV)	Maximum absolute value of the amplitude of the action potential
CV of Amplitude	Coefficient of variation of Amplitude
STV of Amplitude	Short-Term Variance of Amplitude
Area (mm^2^)	Active electrodes area
CV of Area	Coefficient of variation of Area
STV of Area	Short-Term Variance of Area
Velocity	Average local conduction velocity calculated at each electrode location
CV of Velocity	Coefficient of variation of Velocity
STV of Velocity	Short-Term Variance of Velocity
No. of origins	Average number of origins per beat
Distance of origins	Average distance of change in the origins between 2 beats
CV of distance of origins	Coefficient of variation of Distance of origins
STV of distance of origins	Short-Term Variance of Distance of origins

A list of 17 analytical parameters along with detailed descriptions of the respective computational processes involved in their derivation.

The negative control compounds, aspirin and amoxicillin, did not induce significant changes in any of the measured parameters. Among the cardiotoxic compounds, E-4031 significantly reduced beat frequency, field potential amplitude, propagation area, conduction velocity, and the variability in excitation origin locations. Disopyramide produced similar reductions in these parameters and also led to prolonged IBIs. Mexiletine treatment resulted in decreased beat frequency, field potential amplitude, propagation area, and conduction velocity, accompanied by an increase in IBI. Variability in field potential amplitude increased, whereas no significant changes were observed in IBI variability or other variabilities. Azimilide decreased beat frequency, increased IBI, and significantly elevated IBI variability. Isoproterenol increased beat frequency and decreased variability in IBI and propagation area. Diltiazem and Verapamil both increased beat frequency and reduced IBI; Verapamil also increased field potential amplitude and reduced the distance between excitation origins. Quinidine treatment decreased beat frequency, field potential amplitude, and propagation area, while increasing IBI and propagation area variability. Bepridil decreased the variability of IBI, field potential amplitude, and conduction velocity. The parameters that showed statistically significant changes for each compound are summarized in Table 3.

**Table 3.** Summary of quantitative compound effects on analytical parameters.

Compounds	BR	IBI	IBI CV	IBI STV	Amp.	Amp. CV	Amp. STV	Area	Area CV	Area STV	Veloc.	Veloc. CV	Veloc. STV	No. of Origin	Dist Origin	Dist Origin CV	Dist Origin STV
**Diltiazem**	↑	↓	–	–	–	–	–	–	–	–	–	–	–	–	–	–	–
**Isoproterenol**	↑	↓	–	–	–	–	–	–	–	↓	–	–	–	–	–	–	–
**Verapamil**	↑	↓	–	–	–	↓	↓	–	–	–	–	–	–	↓	↓	–	↓
**Azimilide**	↓	↑	–	↑	–	–	–	–	–	–	–	–	–	–	–	–	–
**Disopyramide**	↓	↑	–	–	↓	–	–	↓	–	–	↓	–	–	–	–	–	–
**Mexiletine**	↓	↑	–	–	↓	↑	–	↓	–	–	↓	–	–	–	–	–	–
**E-4031**	↓	–	↑	–	↓	↑	↑	↓	↑	↑	↓	–	–	–	–	↓	–
**Quinidine**	↓	↑	–	–	↓	–	–	↓	↑	–	–	–	–	–	–	–	–
**Bepridil**	–	↓	↓	–	–	↓	–	–	–	–	–	↓	↓	–	–	–	–
**Amoxicillin**	–	–	–	–	–	–	–	–	–	–	–	–	–	–	–	–	–
**Aspirin**	–	–	–	–	–	–	–	–	–	–	–	–	–	–	–	–	–

BR, beat rate; Amp., amplitude; Veloc., velocity.

To evaluate the measurement capabilities of a conventional MEA system, the same set of 7 compounds was tested using the 16-electrode Axion Maestro platform. The results obtained under comparable experimental conditions are summarized in Fig. 5B. Due to the limited spatial resolution of the conventional MEA system, accurate identification of excitation origins was not feasible in many wells. As an alternative, the electrode exhibiting the earliest detected signal was algorithmically designated as the excitation origin for each beat. Consequently, the number of excitation origins was fixed at one by definition, preventing assessment of variability across beats. Furthermore, spatial parameters such as the CV and STV for inter-origin distance and propagation area could not be calculated in 46.5% to 78% of wells, primarily due to baseline values of zero prior to drug application. These parameters are therefore denoted as “—” in the heatmap.

Azimilide elicited consistent reductions in beat frequency and prolongation of IBIs across both systems. A decreasing trend in field potential amplitude was also observed. Although the conventional MEA detected a statistically significant reduction in propagation area, the magnitude of change was less than 5%, suggesting it may fall within physiological variability. Diltiazem produced similar effects in both platforms, including increased beat frequency and shortened IBI. Additionally, a significant reduction in field potential amplitude was detected by the conventional MEA. E-4031 induced consistent reductions in beat frequency, spike amplitude, and propagation area across both platforms. Bepridil induced reductions in IBI variability, amplitude variability, conduction velocity variability, and STV of conduction in CMOS-MEA recordings. However, these changes were not detected by the conventional system. In the conventional MEA, only beat frequency and IBI exhibited statistically significant changes, and their magnitudes remained below 5%, indicating limited physiological relevance. Mexiletine triggered reductions in conduction velocity, propagation area, and amplitude in CMOS-MEA, but these changes were not captured by the conventional MEA. Instead, an increase in spike amplitude was detected, representing an opposite trend. Although changes in beat frequency and IBI reached statistical significance in the conventional system, the magnitude of change was only 2.2%, suggesting it may fall within normal physiological variation. Notably, spontaneous beating ceased entirely at 10 μM in the conventional MEA. Verapamil showed consistent responses across both platforms, including increased beat frequency and shortened IBI. A decreasing trend in spike amplitude was observed in both systems, although statistical significance was not reached in the conventional MEA. Quinidine produced consistent effects across both systems, including reduced beat frequency, prolonged IBI, and decreased amplitude, propagation area, and conduction velocity.

Taken together, the conventional MEA system was able to detect changes in temporal parameters—particularly beat frequency and IBI—that aligned with CMOS-MEA results for 5 of the 7 compounds. However, it failed to detect frequency changes induced by Mexiletine and was unable to record any spontaneous activity at 10 μM.

**Fig. 6.**
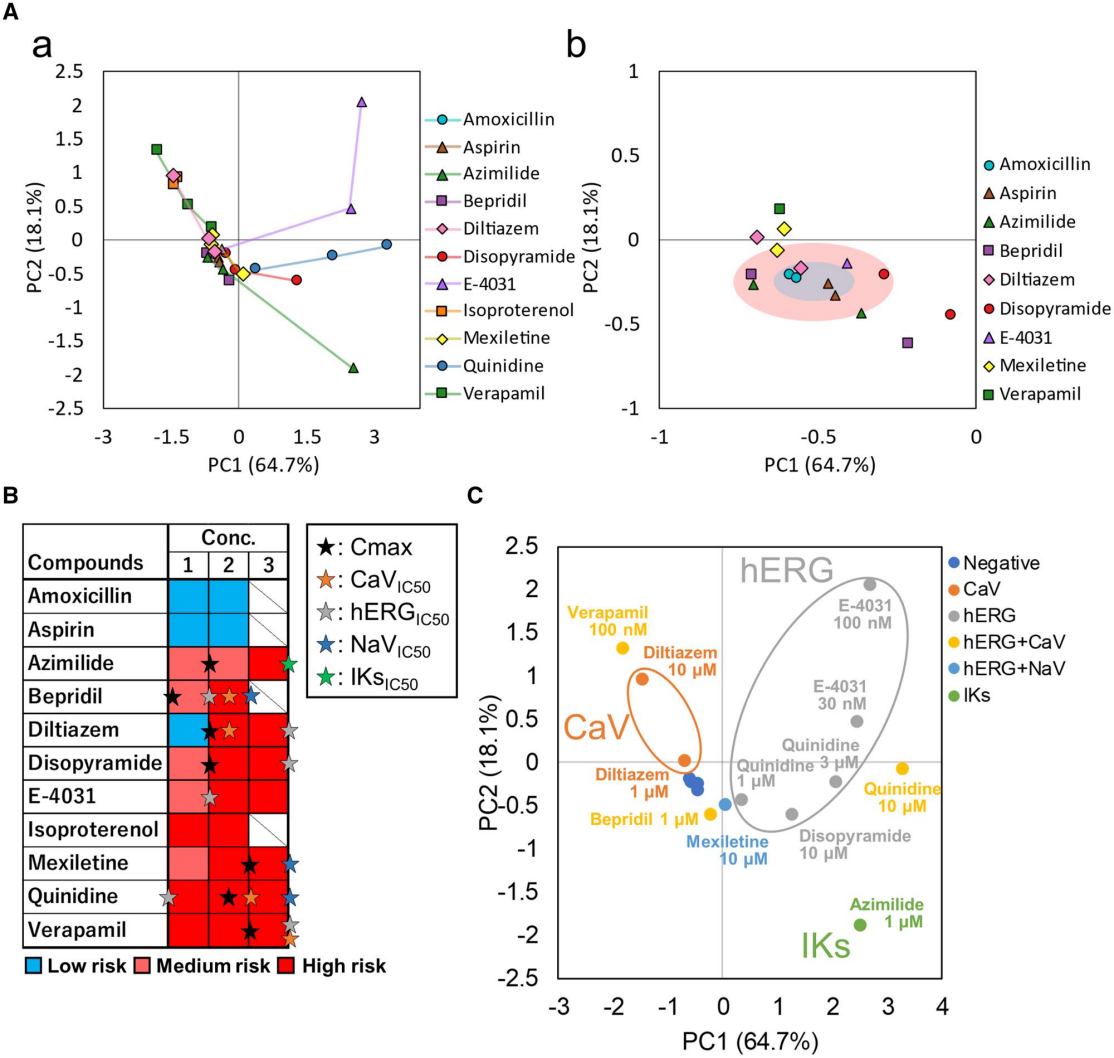
Detection of cardiotoxicity and classification of mechanisms of action using PCA analysis of beat parameters. A) a) PCA plot of compounds. b) Enlarged view of the region near negative control compounds. B) Toxicity classification based on the standard deviation range of negative control compounds. Stars indicate the IC_50_ and Cmax of each ion channel. C) Classification of mechanisms of action on the PCA plot based on IC_50_ values.

To identify the most effective parameter combination for distinguishing cardiotoxic effects, PCA was systematically performed using 65,518 combinations of 2 to 8 parameters selected from the 17 extracted beat-related parameters. Through this exhaustive evaluation, a subset of 4 parameters—beat rate, IBI, propagation area, and STV of conduction velocity—was identified as the optimal parameter set for toxicity classification. This combination was selected based on its ability to maximally separate cardiotoxic from non-cardiotoxic compounds across multiple concentrations. PCA using these 4 representative parameters yielded a cumulative contribution rate of 82.8% for the first 2 PCs (PC1 and PC2), indicating that the majority of variance among samples was captured within a two-dimensional projection space (Table 4). The PCA results for each compound and concentration are presented in Fig. 6A. Negative control compounds (aspirin and amoxicillin) remained near the origin across all tested concentrations. In contrast, cardiotoxic compounds exhibited concentration-dependent displacement away from the origin, forming distinct trajectories in PCA space that reflected their pharmacological profiles. To establish a risk classification framework, the standard deviation (SD) range of negative control compounds in PCA space was used to define 3 zones: Within the SD range (low risk), between 1 and 2 SD (medium risk), and beyond 2 SD (high risk) (Fig. 6B). Risk levels derived from PCA distribution were compared with IC_50_ and Cmax values reported for each compound and associated ion channels (Table 5). All negative controls remained in the low-risk zone at all concentrations. All cardiotoxic compounds reached the high-risk zone at 1 or more tested concentrations.

**Table 4.** Principal component loadings.

	Principal component loadings			
	Beat rate	IBI	Area	Velocity STV
**PC1**	−0.49	0.54	−0.51	0.46
**PC2**	0.60	−0.33	−0.32	0.66

Beat rate, IBI, Area, and STV of Velocity were identified to be an effective parameter set.

**Table 5.** Summary of Cmax free concentrations and IC_50_ values for each ion channel.

Compounds	Cmax Free (μM)	IC_50_ (μM)			
		hERG	NaV	CaV	IKs
**Azimilide**	0.07 (Lee et al. 2025)	5.2 (Busch et al. 1998)	19 (Yao and Tseng 1997)	17.8 (Yao and Tseng 1997)	0.7 (Davies et al. 1996)
**Bepridil**	0.032 (Crumb et al. 2016)	0.16 (Clements et al. 2015)	2.3 (Clements et al. 2015)	1 (Clements et al. 2015)	–
**Diltiazem**	0.128 (Crumb et al. 2016)	13.2 (Kramer et al. 2013)	22.4 (Kramer et al. 2013)	0.76 (Kramer et al. 2013)	–
**Disopyramide**	0.7 (Liu et al. 2021)	14.4 (Kramer et al. 2013)	168.4 (Kramer et al. 2013)	1,037 (Kramer et al. 2013)	–
**E4031**	–	0.014 (Clements et al. 2015)	–	–	–
**Mexiltetine**	2.5 (Crumb et al. 2016)	62.2 (Clements et al. 2015)	38 (Clements et al. 2015)	125 (Clements et al. 2015)	–
**Quinidine**	3 (Blinova et al. 2018)	0.72 (Kramer et al. 2013)	14.6 (Kramer et al. 2013)	6.4 (Kramer et al. 2013)	–
**Verapamil**	0.045 (Crumb et al. 2016)	0.25 (Clements et al. 2015)	32.5 (Clements et al. 2015)	0.2 (Clements et al. 2015)	–

For group-level analysis, compounds were classified into 6 categories based on known ion channel targets: Negative controls, CaV channel inhibitors, hERG channel inhibitors, hERG plus CaV inhibitors, hERG plus NaV inhibitors, and IKs inhibitors. MANOVA was used to assess the separability of these groups (Fig. 6C; Table 6). The Negative, IKs, and hERG plus NaV groups showed significant differences from all other groups. The hERG plus CaV group did not show statistically significant differences from either the hERG or CaV inhibitor groups.

**Table 6.** Statistical analysis of PCA using the effective parameter set (Beat rate, IBI, Area, and STV of Velocity).

	vs. Negative	vs. CaV	vs. hERG	vs. hERG + CaV	vs. hERG + NaV	vs. IKs
**Negative**	–	*P* < 0.001**	*P* < 0.001**	*P* = 0.009**	*P* = 0.001**	*P* < 0.001**
**CaV**	*P* < 0.001**	–	*P* < 0.001**	*P* = 0.177	*P* < 0.001**	*P* < 0.001**
**hERG**	*P* < 0.001**	*P* < 0.001**	–	*P* = 0.326	*P* = 0.043*	*P* < 0.001**
**hERG + CaV**	*P* < 0.009**	*P* = 0.177	*P* = 0.326	–	*P* = 0.044*	*P* < 0.001**
**hERG + NaV**	*P* < 0.001**	*P* < 0.001**	*P* = 0.043*	*P* = 0.044*	–	*P* < 0.001**
**IKs**	*P* < 0.001**	*P* < 0.001**	*P* < 0.001**	*P* < 0.001**	*P* < 0.001**	–

These results describe the quantitative changes in 17 electrophysiological parameters measured by FPI under acute drug treatment conditions. For multivariate analysis, a subset of 4 representative parameters (beat rate, IBI, propagation area, and STV of conduction velocity) was selected for PCA and MANOVA. This analysis provided a basis for evaluating compound-specific toxicity profiles and enabled classification based on known ion channel targets.

### Results 4. Detection of chronic cardiotoxicity using beat parameters

**Fig. 7.**
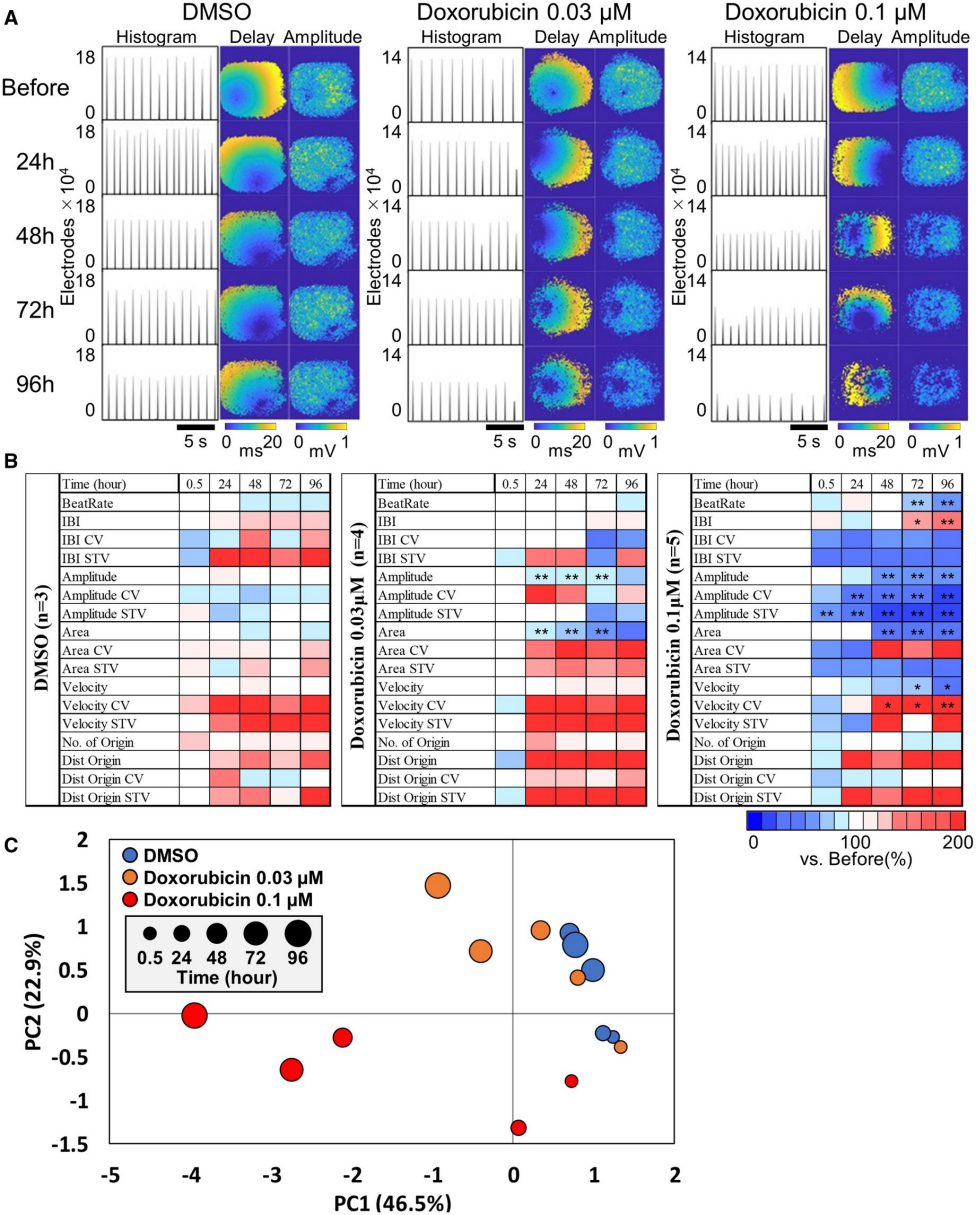
Detection of chronic cardiotoxicity induced by doxorubicin. A) a) Histogram of the number of active electrodes before and after chronic treatment (left), heatmap of peak timing for a single beat (middle), and heatmap of peak amplitude for a single beat (right). B) Time-dependent heatmap of beat parameters after chronic treatment. One-way ANOVA followed by Dunnett’s post hoc test was performed. *P < 0.05 vs. before, **P < 0.01 vs. before. C) PCA plot after chronic treatment.

To validate the chronic cardiotoxicity assessment using CMOS-MEA measurements, FPI was performed following long-term exposure to doxorubicin. Doxorubicin was administered at 0.03 and 0.1 µM for 96 h, with DMSO used as a vehicle control. Recordings were acquired before exposure and at 24-h intervals. Figure 7A displays representative results, including histograms of active electrodes, delay maps, and amplitude maps. Long-term DMSO exposure resulted in no significant temporal changes in beat rate, beat area, or peak potential. In contrast, 0.03 µM doxorubicin did not significantly alter beat rate but led to significant decreases in beat area and peak potential after 24 h. At 0.1 µM, reductions in beat rate, beat area, and peak potential were observed beginning at 48 h. Detailed time-course changes in beat-related parameters are visualized in the heatmap shown in Fig. 7B. During chronic DMSO exposure, minor changes were observed in the CV and STV, but these were not statistically significant. At 0.03 µM doxorubicin, beat area and peak potential decreased significantly after 24 h. At 0.1 µM, STV of peak potential decreased within 0.5 h, followed by a reduction in CV after 24 h. After 48 h, the CV of conduction velocity increased along with continued reductions in beat area and peak potential. Changes in conventional parameters such as beat rate and IBI became evident only after 72 h.

PCA was conducted to integrate multiple parameter changes into a comprehensive index of cardiotoxicity. The PCA included 6 selected parameters: IBI, Amplitude, Amplitude STV, Area, Velocity, and Velocity STV (Table 7). The first 2 PCs accounted for a cumulative contribution of 69.4%. The results are shown in Fig. 7C. DMSO-treated samples exhibited minimal shifts over time, whereas both concentrations of doxorubicin showed progressive, concentration-dependent displacement in PCA space. For statistical analysis of cardiotoxicity across time points, PCA plots were categorized into 15 groups based on concentration and exposure duration. MANOVA was performed to compare each group against the DMSO 0.5 h reference (Table 8). No significant differences were detected among DMSO time points. At 0.03 µM doxorubicin, significant differences emerged after 24 h. At 0.1 µM, significant differences were observed after 24 h relative to all DMSO time points.

**Table 7.** Principal component loadings.

	Principal component loadings					
	IBI	Amplitude	Amplitude STV	Area	Velocity	Velocity STV
**PC1**	−0.32	0.47	0.46	0.52	0.43	−0.10
**PC2**	0.27	−0.07	0.42	−0.21	0.26	0.79

IBI, Amplitude, STV of Amplitude, Area, Velocity, and STV of Velocity were identified to be an effective parameter set to remove the effect of time course of DMSO exposure.

**Table 8.** Statistical analysis of PCA using the effective parameter set (IBI, Amplitude, Amplitude STV, Area, Velocity, and Velocity STV) to eliminate the effect of time course of DMSO exposure.

	DMSO					Doxorubicin 0.03 μM					Doxorubicin 0.1 μM				
	0.5 h	24 h	48 h	72 h	96 h	0.5 h	24 h	48 h	72 h	96 h	0.5 h	24 h	48 h	72 h	96 h
**DMSO 0.5 h**	–	*P* = 0.987	*P* = 0.511	*P* = 0.489	*P* = 0.267	*P* = 0.924	*P* = 0.932	*P* = 0.471	*P* = 0.445	*P* = 0.300	*P* = 0.554	*P* = 0.382	*P* = 0.319	*P* = 0.315	*P* = 0.066
**DMSO 24 h**	*P* = 0.987	–	*P* = 0.684	*P* = 0.802	*P* = 0.397	*P* = 0.047*	*P* = 0.641	*P* = 0.813	*P* = 0.964	*P* = 0.828	*P* = 0.033*	*P* = 0.014*	*P* = 0.016*	*P* = 0.029*	*P* = 0.001**
**DMSO 48 h**	*P* = 0.511	*P* = 0.684	–	*P* = 0.904	*P* = 0.989	*P* = 0.024*	*P* = 0.227	*P* = 0.821	*P* = 0.341	*P* = 0.567	*P* = 0.002**	*P* = 0.001**	*P* = 0.004**	*P* = 0.006**	*P* = 0.002**
**DMSO 72 h**	*P* = 0.489	*P* = 0.802	*P* = 0.904	–	*P* = 0.917	*P* = 0.030*	*P* = 0.135	*P* = 0.373	*P* = 0.187	*P* = 0.216	*P* = 0.008**	*P* = 0.002**	*P* = 0.008**	*P* = 0.018*	*P* = 0.001**
**DMSO 96 h**	*P* = 0.267	*P* = 0.397	*P* = 0.989	*P* = 0.917	–	*P* = 0.017*	p = 0.060	p = 0.168	p = 0.076	p = 0.080	*P* = 0.004**	*P* < 0.001**	*P* = 0.005**	*P* = 0.012*	*P* < 0.001**

*P < 0.05 vs. corresponding time point in DMSO group.

**P < 0.01 vs. corresponding time point in DMSO group.

Bold values indicate statistically significant comparisons.

These results describe the time-dependent alterations in electrophysiological parameters following chronic doxorubicin exposure and demonstrate the ability of FPI-based analysis to quantify these changes using multivariate approaches.

## Discussion

FPI using CMOS-MEA offers high-resolution measurements capable of simultaneously recording field potentials from a single cell across multiple electrodes. This allows for the analysis of the electrophysiological activity of entire hiPSC-CM networks with high spatial and temporal resolution (Fig. 1). In this study, we introduced several spatially resolved parameters—such as the number of excitation origins, propagation velocity, and propagation area—that were difficult to quantify with sufficient precision using conventional MEA systems. These parameters proved useful for characterizing compound-specific responses and suggesting potential mechanisms of action.

Specifically, in addition to an increase in beating frequency, an increase in the number of excitation origins was observed with Isoproterenol. This suggests that Isoproterenol, a β-adrenergic agonist, captures the characteristic arrhythmogenic effects in cardiomyocytes, including the induction of early afterdepolarizations (EADs) and/or delayed afterdepolarizations (Fig. 2) (Zeng and Rudy 1995; Paci et al. 2020). With Mexiletine, a decrease in beating frequency was observed at 10 μM, indicating that the bradycardic effect of Mexiletine was also detected in MEA measurements. Furthermore, a reduction in propagation velocity was observed, suggesting that the restricted influx of sodium ions delays the propagation of ionic excitation between cells (Fig. 3). For E-4031, a decrease in propagation area and the detection of EAD-like waveforms were observed, revealing conduction abnormalities that reflect the arrhythmia risk associated with hERG channel inhibition (Fig. 4). The novel parameters derived from FPI serve as effective endpoints that reflect mechanisms of action and have the potential to establish FPI as a next-generation myocardial MEA assay for predicting drug-induced cardiotoxicity risks. Amplitude-related parameters also showed significant changes in the acute response of many compounds (Fig. 5A), and doxorubicin in chronic treatment assays also exhibited prominent changes. This indicates that the amplitude calculated by the UHD-CMOS-MEA system, derived from over 100,000 active electrodes capable of recording the activity of the entire cell population, exhibits higher sensitivity in detection. Comparison of cardiotoxicity detection using novel FPI endpoints and conventional methods revealed that changes induced by Mexiletine were detected at lower concentrations than in traditional assays. Furthermore, FPI enabled the detection of changes induced by Bepridil, which were difficult to capture using conventional MEA measurements (Fig. 5, Table 9), demonstrating its potential contribution to advancing in vitro cardiotoxicity assessment.

**Table 9.** Comparison with cardiotoxicity assay benchmark.

Drug	Tested conc. (μM)	MEA assay (µM)	Impedance assay (µM)	HCA assay (µM)	FPI (μM)	FPI PCA (μM)
Bepridil	0.1, 1	10 (Kaneko et al. 2014) (FPD)	0.03 (Guo et al. 2013) (BR20)	1.0 (Lu et al. 2019) (CTD90 ↑)	0.1 (Amp. CV, Veloc. CV, Veloc. STV)	1
Azimilide	0.01, 0.1, 1	0.129 (Nozaki et al. 2017) (FPDc10)		1.0 (Lu et al. 2019) (CTD90 ↑)	1 (BR, IBI, IBI STV)	1
Diltiazem	0.1, 1, 10	0.300 (Nozaki et al. 2014) (FPDc10)	0.1 (Guo et al. 2013) (BR20)	0.1 (Lu et al. 2019) (CTD90 ↓)	1 (BR, IBI)	1
Disopyramide	0.1, 1, 10	0.74 (Ando et al. 2017) (FPDc10)		10 (Lu et al. 2019) (CTD90 ↑)	1 (BR)	1
Isoproterenol	0.01, 0.03	N/A (Nozaki et al. 2017)	0.01 (Doherty et al. 2015) (BR20)		0.01 (BR, IBI)	0.01
Quinidine	1, 3, 10	0.3 (Harris et al. 2013) (FPD)	10 (Guo et al. 2013) (BR20)	N/A (Dempsey et al. 2016)	1 (BR)	1
E-4031	0.003, 0.03, 0.1	0.003 (Clements and Thomas 2014) (FPD20)	0.03 (Guo et al. 2013) (BR20, IB20)	0.03 (Aikawa 2017) (PWD10)	0.03 (BR, Area)	0.03
Mexiletine	0.1, 1, 10	3.0 (Clements and Thomas 2014) (Spike amp.)	3.0 (Guo et al. 2013) (BR20)		10 (BR, IBI, Ampl. Area, Veloc.)	1
Verapamil	0.003, 0.03, 0.1	0.01 (Clements and Thomas 2014) (FPD20)	0.03 (Guo et al. 2013) (BR20)	0.1 (Dempsey et al. 2016) (Ca^2+^ amp.20↓)	0.003 (IBI, Amp. STV, Dist Origins, Dist Origins STV)	0.003

FPD, filed potential duration; FPD20, 20% increase in FPD; FPDc, corrected FPD; FPDc10, 10% prolongation of FPDc; Spike amp., spike amplitude; BR20, 20% reduction in beat rate; IB20, 20% increase in irregular beats; CTD90, calcium transient duration at 90% of decay following the peak amplitude; PWD, calusium fluorescence peak width; Ca2+ amp.20, 10% decreasee of calcium waveforms amplitude.

To further demonstrate the advantages of the UHD-CMOS-MEA system, a comparative analysis was performed using the same set of 7 compounds and a conventional MEA system based on the 16-electrode Axion Maestro platform (Fig. 5B). Although changes in beat frequency and IBI were also detected by the conventional MEA, its limited spatial resolution made it difficult to quantify spatial parameters, such as the number of excitation origins, their positional variability, and fluctuations in propagation area. In particular, variability indices—such as the CV and STV for inter-origin distance and propagation area—could not be calculated in more than 46% of wells due to zero baseline values prior to compound administration.

Reductions in conduction velocity and propagation area induced by Mexiletine were clearly identified using the CMOS-MEA but were not detected as significant changes with the conventional MEA. Moreover, spontaneous beating ceased entirely at 10 μM in the conventional system. This outcome may be attributed to the marked reduction in propagation area observed with the CMOS-MEA, which the lower spatial resolution of the conventional MEA may have failed to recognize as valid beating activity.

Furthermore, a direct comparison was conducted between the UHD-CMOS-MEA and conventional MEA systems under chronic exposure to 0.1 μM doxorubicin. Although the conventional MEA began to detect a reduction in spike amplitude at 48 h, the CMOS-MEA detected a decline in conduction velocity as early as 24 h (Fig. S1). These findings highlight the superior spatiotemporal resolution and sensitivity of FPI in capturing subtle and progressive electrophysiological deterioration, reinforcing its value in chronic cardiotoxicity assessment.

The results of PCA using the novel endpoints—Area and Velocity STV—along with Beat rate and IBI, demonstrated the ability to clearly capture concentration-dependent changes and evaluate complex toxic effects, including Bepridil, which was difficult to detect using conventional MEA measurements and is classified as an outlier compound in CiPA (Fig. 6A). Bepridil is a hERG blocker with a tendency to prolong repolarization; however, due to its additional properties as a Ca^2+^ and Na^+^ channel antagonist, the extent of repolarization prolongation tends to be counterbalanced, making cardiotoxicity detection challenging (Blinova et al. 2018). The multivariate analysis incorporating novel endpoints, combined with an evaluation method based on the SD range of negative controls, suggests its effectiveness as a cardiotoxicity assessment method (Fig. 6A). Compounds acting on multiple ion channels exert their combined effects on cells and tissues. Conventional IC_50_-based toxicity risk assessments, particularly those focused on hERG inhibition, have faced challenges due to discrepancies between in vitro assessments and clinically toxic doses (Li et al. 2017; Romero et al. 2018). hiPSC-derived cardiomyocyte assays, which inherently possess multiple ion channels, enable simultaneous multi-channel evaluation (Lee et al. 2024). The FPI technology and multivariate analysis incorporating novel endpoints enable toxicity assessment that accounts for multichannel effects, contributing to improved accuracy in clinical toxicity prediction.

Furthermore, in the classification of drug mechanisms of action using PCA, compound groups with a single mechanism, such as those acting on hERG, Na, and Ca channels, were significantly separated, demonstrating that mechanism-based prediction is feasible (Table 6). Additionally, the classification of compounds acting on multiple channels was examined. As a result, Diltiazem 10 μM (hERG: 76%, NaV: 45%, CaV: 1316%, with each value representing the ratio relative to IC_50_) was plotted near compounds acting on CaV channels (Fig. 6C). Similarly, Quinidine 10 μM (hERG: 1389%, NaV: 68%, CaV: 156%, with each value representing the ratio relative to IC_50_) was plotted near compounds acting on hERG channels (Fig. 6C). The coordinates of the PCA plot reflected differences in IC_50_ values across channels for each compound, demonstrating that even when a compound acts on multiple channels, its predominant mechanism of action can still be evaluated.

Moreover, these findings suggest that the combination of FPI and PCA analyses contributes not only to cardiotoxicity risk assessment but also to the elucidation of mechanisms of action (Fig. 6). Predicting mechanisms of action can facilitate lead compound optimization in the early stages of drug discovery and is also expected to be useful for predicting off-target effects.

In our chronic cardiotoxicity assessment of doxorubicin, significant electrophysiological alterations were detected at a low concentration (0.03 μM) and within a short exposure period (24 h). Specifically, early reductions in propagation area and peak field potential amplitude were observed in the absence of changes in beating frequency, suggesting the onset of electrical desynchronization and tissue-level remodeling (Fig. 7A and B). At a higher concentration (0.1 μM), prolonged exposure (48 h) further induced a decrease in conduction velocity and an increase in temporal variability metrics (CV and STV), indicating progressive disruption of propagation synchrony.

These findings stand in contrast to previous reports using conventional platforms. For example, impaired Ca^2+^ handling and reduced contractility in mouse cardiomyocytes have been observed only after treatment with 1 μM doxorubicin (Lin et al. 2024). Similarly, studies in rat cardiomyocytes demonstrated that doxorubicin-induced Ca^2+^ handling abnormalities triggered apoptosis and contractile dysfunction, but only at micromolar concentrations (Sag et al. 2011; Ikeda et al. 2019). Engineered heart tissues derived from human iPSCs also exhibited reduced contractile force only after 48 h of exposure to 1 μM doxorubicin, with no observable effects at lower concentrations of 125 or 500 nM (Arefin et al. 2023).

Furthermore, in a study utilizing deep learning-based morphological profiling of monolayer hiPSC-CMs, structural changes were only detected at concentrations exceeding 0.3 μM (Maddah et al. 2020). Compared with these prior studies, our UHD-CMOS-MEA platform detected doxorubicin-induced cardiotoxicity at a 10-fold lower concentration (0.03 μM) and within a shorter timeframe (24 h).

These early electrophysiological abnormalities—specifically, reductions in propagation area and peak field potential amplitude induced by doxorubicin—were captured with high spatial resolution and likely reflect early disruptions in Ca^2+^ handling and electrical synchrony. Notably, propagation area changes were not detectable using conventional methods. Taken together, these findings demonstrate that the UHD-CMOS-MEA enables earlier and more sensitive detection of chronic cardiotoxicity than previously possible with established in vitro models. This enhanced sensitivity is attributed to the platform’s ability to detect subtle electrophysiological disturbances that precede structural or functional deterioration, offering a powerful tool for preclinical cardiac safety evaluation.

Electrical pacing is commonly employed in conventional MEA systems to control the beating rate and minimize variability in rate-dependent electrophysiological parameters, such as beat interval, conduction velocity, and field potential amplitude. In contrast, all measurements in the present study using the UHD-CMOS-MEA system were conducted under spontaneous beating conditions. As a result, fluctuations in the spontaneous beating rate between wells may have indirectly influenced rate-dependent endpoints—e.g. a faster beating rate could result in shortened conduction velocity or reduced field potential amplitude due to incomplete ion channel recovery. This potential source of variability should be considered when interpreting the data. To address this limitation, the UHD-CMOS-MEA platform is currently being upgraded to incorporate an electrical stimulation capability. Once implemented, this feature will enable external pacing in future experiments, allowing for more precise control of rate-dependent electrophysiological parameters.

The novel endpoints derived from FPI using the UHD-CMOS-MEA system, which provides high spatial and temporal resolution—such as the number of excitation origins, conduction velocity, and propagation area—combined with multivariate analysis for cardiotoxicity assessment, represent a next-generation in vitro cardiotoxicity prediction platform. This platform is expected to enable not only the risk assessment of cardiotoxicity across a broad range of drug candidates but also the elucidation of mechanisms of action and the prediction of off-target effects.

## Supplementary Material

kfaf134_Supplementary_Data
