# Supplementary material for: Advanced cardiotoxicity profiling using field potential imaging with UHD-CMOS-MEA in human iPSC-derived cardiomyocytes
Source: Toxicol Sci. 2025 Sep 30;208(2):384–400. doi: 10.1093/toxsci/kfaf134 (PMC12646587; doi:10.1093/toxsci/kfaf134)
Supplement: kfaf134_Supplementary_Data [file kfaf134_supplementary_data.zip › kfaf134_Supplementary_Data/toxsci-25-0276-File009.docx]

**Supplementary material**

**Advanced Cardiotoxicity Profiling Using Field Potential Imaging with UHD-CMOS-MEA in Human iPSC-Derived Cardiomyocytes**

**Authors**

Naoki Matsuda^†1^, Nami Nagafuku^†1^, Kazuki Matsuda^1^, Yuto Ishibashi^1^, Tomohiko Taniguchi^2^, Yusaku Matsushita^2^, Norimasa Miyamoto^2^, Takashi Yoshinaga^2^, Ikuro Suzuki**^*^**^1^

**Affiliations**

^1^Department of Electronics, Graduate School of Engineering, Tohoku Institute of Technology, 35-1 Yagiyama Kasumicho, Taihaku-ku, Sendai, Miyagi, 982-8577, Japan

^2^Advanced Biosignal Safety Assessment, Biopharmaceutical Assessments Unit, Eisai Co., Ltd., 5-1-3 Tokodai, Tsukuba, Ibaraki 300-2635, Japan

**^＊^Corresponding author:**

Ikuro Suzuki

Tel: +81-22-305-3219

Fax: +81-22-305-3219

E-mail: [i-suzuki@tohtech.ac.jp](mailto:i-suzuki@tohtech.ac.jp)


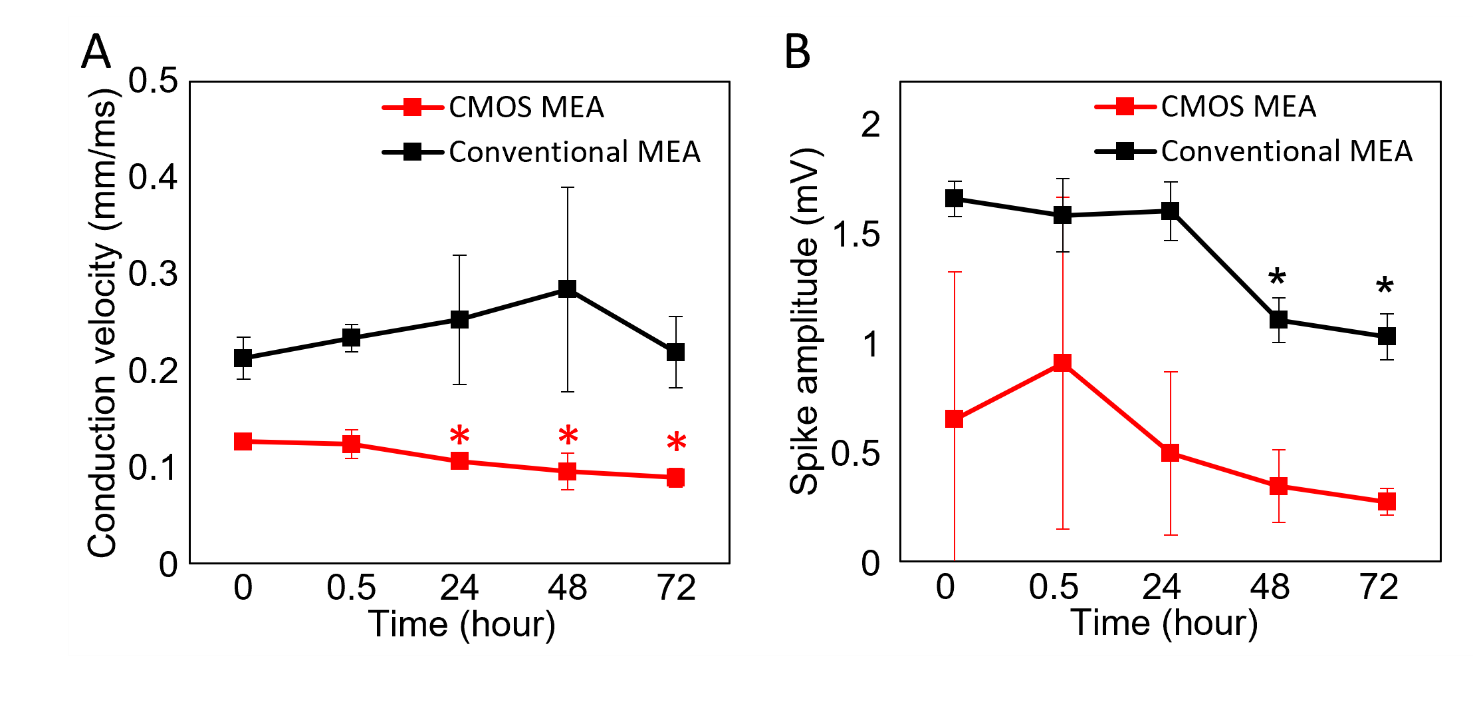


**Supplementary Figure 1. Comparison of raw parameter values between conventional MEA and CMOS-MEA systems following chronic exposure to 0.1 μM doxorubicin.**
(A) Time course of conduction velocity is shown for both conventional MEA (n = 4) and CMOS-MEA (n = 5).

(B) Time course of spike amplitude is presented under the same conditions.

Data are presented as raw values (mean ± SD). *p < 0.05 vs. baseline. Statistical significance was assessed using one-way ANOVA followed by Dunnett’s post hoc test.

**Supplementary movie Legends**

**Supplementary movie 1**

**Spatiotemporal propagation of a single field potential wave in human iPSC-derived cardiomyocytes cultured on a CMOS microelectrode array.**

This movie visualizes the field potential propagation during one spontaneous beat recorded from human iPSC-derived cardiomyocyte monolayers using an ultra-high-density CMOS-MEA platform. The sampling rate was 2 kHz, and the data are presented at 10 FPS.

**Supplementary movie 2**

**Comparison of field potential propagation before and after administration of isoproterenol 30 nM in human iPSC-derived cardiomyocytes cultured on a CMOS microelectrode array.**

This video shows the spatiotemporal propagation of field potentials captured during a single beat. The left panel shows baseline activity prior to drug administration, while the right panel presents the activity following treatment with 30 nM Isoproterenol. An increase in the number of excitation origins is clearly observed following isoproterenol exposure. The data were acquired at a sampling rate of 2 kHz and displayed at 10 frames per second. The color scale represents field potential amplitude, and the time displayed at the top of the video indicates the elapsed time from the onset of the beat.

**Supplementary movie 3**

**Field potential propagation before and after administration of mexiletine (10 μM) in human iPSC-derived cardiomyocytes cultured on a CMOS microelectrode array.**

This video shows the spatiotemporal propagation of field potentials during a single cardiac beat. The left panel shows baseline activity prior to drug administration, while the right panel presents the activity following treatment with 10 μM mexiletine. A clear reduction in both conduction velocity and field potential amplitude is observed after drug exposure. Data were recorded at a sampling rate of 2 kHz and are displayed at 10 frames per second. The color scale indicates field potential amplitude, and the time shown at the top of the video represents the elapsed time from the onset of the beat.

**Supplementary movie 4**

**Propagation of EAD-like waveforms and arrhythmic activity in human iPSC-derived cardiomyocytes following E-4031 exposure.**

This video presents four representative spatiotemporal propagation patterns of field potentials recorded from human iPSC-derived cardiomyocytes cultured on a CMOS microelectrode array after treatment with the hERG channel inhibitor E-4031 (30 nM).

The leftmost panel displays a typical beat with high amplitude and widespread propagation. The second panel depicts the propagation of EAD-like waveforms, characterized by localized, low-amplitude signals, indicative of early afterdepolarization (EAD)-like activity. The third and fourth panels show arrhythmic propagation, occurring shortly after the EAD-like events, exhibiting irregular and focal conduction patterns.

Data were acquired at a sampling rate of 2 kHz and are displayed at 10 frames per second. The color scale represents field potential amplitude, and the time axis at the top of each panel indicates the elapsed time from the onset of the beat.
